# Polyphenols as modulators of pre‐established gut microbiota dysbiosis: State‐of‐the‐art

**DOI:** 10.1002/biof.1772

**Published:** 2021-08-16

**Authors:** Romina Molinari, Nicolò Merendino, Lara Costantini

**Affiliations:** ^1^ Department of Ecological and Biological sciences (DEB) Tuscia University, Largo dell'Università snc Viterbo Italy

**Keywords:** dietary polyphenols, dysbiosis‐related diseases, flavonoids, gut microbiota, proanthocyanidins

## Abstract

The human intestine contains an intricate ecological community of bacteria, referred as the gut microbiota, which plays a pivotal role in the host homeostasis. Multiple factors could interfere with this delicate balance, thus causing a disruption of the microbiota equilibrium, the so called dysbiosis. Gut microbiota dysbiosis is involved in gastrointestinal and extra‐intestinal metabolic diseases, as obesity and diabetes. Polyphenols, present in a broad range of plant foods, are known to have numerous health benefits; however, their beneficial effect on pre‐existing dysbiosis is less clear. Indeed, in most of the conducted animal studies the administration of polyphenols or foods rich in polyphenols occurred simultaneously with the induction of the pathology to be examined, then analyzing the preventive action of the polyphenols on the onset of dysbiosis, while very low studies analyzed the modulatory activity of polyphenols on the pre‐existing dysbiosis. For this reason, the present review aims to update the current information about the modulation of the pre‐established gut microbiota dysbiosis by dietary phenolic compounds in a broad range of disorders in both animal studies and human trials, distinguishing the preventive or treatment approaches in animal studies. The described studies highlight that dietary polyphenols, exerting prebiotic‐like effects, can modulate the pre‐existing dysbiosis stimulating the growth of beneficial bacteria and inhibiting pathogenic bacteria in both animal models and humans. Anyway, most of the conducted studies are related to obesity and metabolic syndrome, and so further studies are needed to understand this polyphenols' ability in relation to other pathologies.

**Abbreviations**
ALT: alanine aminotransferase
APE: apple polyphenol extract
ApoE^−/−^
: Apolipoprotein E
AST: aspartate aminotransferase
BMI: body mass index
BW: body weight
CD: Crohn disease
CRC: colorectal cancer
CRP: c reactive protein
CTR: control
DGGE: denaturing gradient gel electrophoresis
DP: degree of polymerization
DSS: dextran sulphate sodium
EGCG: epigallocatechin gallate
EGCG3‐Me: epigallocatechin 3‐*O*‐(3‐*O*‐methyl) gallate
F: fraction
*F/B*
: *Firmicutes/Bacteroidetes*
GMCSF: granulocyte macrophage colony‐stimulating factor
GRO: growth‐regulated oncogene
GSPE: grape seed proanthocyanidin extract
GTE: green tea extract
HbA1c: hemoglobin A1c
HFD: high‐fat diets
HFHSD: high‐fat high‐sucrose‐diet
HTS: high throughput sequencing
IBDs: inflammatory bowel diseases
ISAPP: International Scientific Association for Probiotics and Prebiotics
LDLR^−/−^
: LDL receptor‐deficient
LFD: low‐fat diet
LPS: lipopolysaccharide
MCD: methionine‐choline deficient
Mets: metabolic syndrome
NAFLD: nonalcoholic fatty liver disease
NASH: nonalcoholic steatohepatitis
PACs: oligomeric proanthocyanidins
PCR‐DGGE: polymerase chain reaction‐denaturing gradient gel electrophoresis
PFE: *Pyracantha fortuneana* fruit extract
PPEP: peel peach extracted polyphenols
SASP: sulfasalazine
SCFAs: short‐chain fatty acids
TLR4: Toll like receptor 4
TMAO: trimethylamine‐N‐oxide
TNBS: 2,4,6‐trinitrobenzenesulfonic acid
TPC: total polyphenols' content
UC: ulcerative colitis
w/v: weight/volume

## 1 INTRODUCTION

The gastrointestinal tract is colonized by about 100 trillion of microorganisms, consisting mainly of bacteria and to a lesser extent by viruses, protozoa, and yeast.^1^ This community of microorganisms living on this site are described with the term of gut microbiota and the abundance, type, and function of the different microorganisms differ along the gastrointestinal tract. However, even though some microorganisms can be present long the entire intestine, most are found in the large intestine, where these bacteria participate in the transformation of undigested food components (carbohydrates, lipids, and proteins) regulating lipid metabolism and glucose homeostasis and leading to the formation of short‐chain fatty acids (SCFAs).^2^ Taxonomically, bacteria are classified according to phyla, class, order, family, genera, and species. The predominant phyla in the gut are *Firmicutes*, *Bacteroidetes*, *Actinobacteria*, *Proteobacteria*, *Fusobacteria*, and *Verrucomicrobia*, but 90% of the gut population is made up of *Firmicutes* and *Bacteroidetes*.^3^ There is growing evidence that perturbations in the gut microbiota composition (dysbiosis) are associated with a reduced diversity and with the overgrowth of few pathogenic and pathobiont taxa, and these events can be closely linked to many human diseases. Dysbiosis depends on various factors such as age, diet, lack of exercise, stress, intake of drugs, and xenobiotics, and the ratio of *Firmicutes*/*Bacteroidetes* (*F/B*) is a rough indicator of a microbial composition shift. Since the intestinal microbiota plays a central role in many crucial mechanisms for the physiology and metabolism of the host, dysbiosis can be associated not only with intestinal disorders such as inflammatory bowel diseases (IBDs), ulcerative colitis (UC), Crohn's disease (CD), and colorectal cancer (CRC), but also with various extra‐intestinal diseases such as cardiovascular and metabolic disorders (obesity and diabetes) and neurological diseases.^4^ Polyphenols and other phenolic compounds are secondary plant metabolites and can be further divided into classes according to the number of phenolic rings and the bonds that join the rings. These compounds are generically classified as flavonoids and no‐flavonoids. Flavonoids can be divided into various subgroups including flavones, flavonols, and flavan‐3‐ols such as epigallocatechin gallate (EGCG), isoflavones, flavanones, and anthocyanidins. The no‐flavonoid group includes phenolic acids such as caffeic acid, lignans, and stilbenes, among which resveratrol.^5^ Dietary sources of polyphenols include berries, grapes and wine, tea, chocolate, coffee, and a variety of fruits and vegetables. Several studies have focused on the effects of various dietary polyphenols in the prevention and treatment of multiple chronic disorders, such as cardiovascular disease, type II diabetes, obesity, arthritis, IBDs, Alzheimer's, and Parkinson's diseases.^6^ Initially, the beneficial effects of polyphenolic compounds were attributed to their antioxidant capacity able to inactivate reactive oxidant species. Instead, recent studies are showing that the beneficial effects are also related with the ability of polyphenols to interact with the main cell signaling and gene regulation pathways and to the modulation of the gut microbiota.^7^ Indeed, for example, polyphenols can influence the *F/B* ratio through growth inhibition of specific bacterial species.^8^ Here, we have reviewed the recent studies concerning the ability of pure polyphenols and polyphenol‐rich foods to modulate the dysbiosis present in various diseases.

## 2 EFFECTS OF PURE POLYPHENOLS' COMPOUNDS ON THE GUT MICROBIOTA DYSBIOSIS

Recent studies have shown that polyphenols can alter the intestinal microbiota, decreasing systemic inflammation, and improving metabolic responses in subjects with pathologies.^9^, ^10^ Polyphenols may also exert beneficial effects on the microbiota by acting similarly to prebiotics; in fact, in 2017 the ISAPP (International Scientific Association for Probiotics and Prebiotics) added the phenolics/phytochemicals between the “candidates prebiotics” and defining the prebiotics as “a substrate that is selectively utilized by host microorganism conferring a health benefit.”^11^ They indirectly prompted the researchers to implement the scientific evidence of polyphenols as prebiotics for the confirmation of this title as for other accepted and well‐known prebiotics (e.g., fructans and galactans), and this evidence is described below. In particular, here the analysis of literature concerning the pure compounds or polyphenol‐rich extracts (i.e., extract mostly rich in a specific phenolic compound) on the gut microbiota were analyzed. Differently, in the Section 3, the studies concerning the action of polyphenols on gut microbiota administered as whole food matrix have been discussed. Animal studies and clinical trials were differently treated. Anyway, it is noteworthy that in most of the animal models studies the inducer of the pathology and the treatment with polyphenols was administered at the same time, thus analyzing mostly the polyphenols preventive action. In order to provide an overall framework, these studies were analyzed below, while the polyphenols' effects on pre‐established dysbiosis will be analyzed in the Section 2.1.2.

### 2.1 Animal model studies

#### 2.1.1 Animal studies on the preventive action of pure polyphenols on the onset of dysbiosis

IBDs are debilitating chronic inflammatory disorders whose onset is associated with dysbiosis, a situation that persists even in patients in remission. The origins include genetic predisposition and environmental components such as exposure to antibiotics and diet.^12^ IBDs include two major disorders: ulcerative colitis and Crohn's disease, both characterized by chronic intestinal inflammation.^13^ Several studies have shown how the use of polyphenols can help to correct the intestinal dysbiosis characteristic of IBDs. Polyphenols can exert health effects through modulation of the gut microbiota composition when they are supplied in the diet as pure compounds or as polyphenol‐rich extracts. Liu and co‐workers^14^ investigated the preventive effects of various concentrations of apple polyphenol extract (APE) with phloretin (mostly present polyphenol in apples) on dextran sulphate sodium (DSS)‐induced ulcerative colitis in male mice. For this purpose, male C57BL/6 mice were treated with 125 or 500 mg kg^−1^ body weight (BW) at day of APE or 100 mg kg^−1^ BW at day of phloretin for 2 weeks by intragastric administration, and then with both APE or phloretin and 3% (w/v) DSS dissolved in drinking water for 1 week to induce ulcerative colitis. The results showed that APE and phloretin were not able to improve the reduction of microbiota diversity caused by DSS; on the contrary they exacerbated this reduction, probably because the polyphenols had an antibacterial activity, which could inhibit the growth of many harmful bacteria. However, a significant increased abundance of *Verrucomicrobia* after APE intervention and significant decreased abundance of *Firmicutes* after phloretin intervention was observed. APE and phloretin intervention induced increase of genus *Bacteroides* and *Akkermansia* compared to DSS group. Despite the interesting results, this work has a limit: mice have free access to water containing 3% weight/volume (w/v) DSS and this could lead to a different consumption, which could then be reflected in an inconsistent development of ulcerative colitis. These results were partly confirmed by the study of Liu and collaborators^15^ where Kunming female mice were fed different types of tea extracts. DSS‐treated mice were fed with 200 μl of single tea extracts at a concentration of 0.5 mg ml^−1^ (5 mg kg^−1^ BW), starting from day 1 to day 14. The colitis model was established by administrating 3% (w/v) DSS water, and mice received 10 ml kg^−1^ BW water (for health and DSS group) or the same volume of 0.05% (w/v) tea extract solution (for tea extracts‐treated groups). The authors concluded that the treatment of various types of tea can alleviate DSS‐induced colitis in mice and modulate the dysbiosis of gut microbiota. Indeed, tea extracts had attenuated the DSS‐induced decrease in the richness and diversity of gut microbiota, and they had increased the abundance of the beneficial bacteria (e.g., *Faecalibaculum* and *Bifidobacterium*), and decreased the abundance of the harmful bacteria (e.g., *Bacteroides* and *Mucispirillum*). Another study investigated whether resveratrol, a polyphenol found in a variety of foods and drinks, could reverse the microbial dysbiosis induced during colitis. For this purpose, the oxidizing agent 2,4,6‐trinitrobenzenesulfonic acid (TNBS) was administered intrarectally one time to female BALB/c mice at a dose of 1 mg; resveratrol (100 mg kg^−1^) was given daily, 24 h prior to TNBS injection until completion of the experiment (5 days). Analysis of stools revealed that TNBS administration increased species such as *Bacteroides acidifaciens* and decreased species such as *Ruminococcus gnavus* and *Akkermansia muciniphila*; *Ruminococcus gnavus* and *Akkermansia muciniphila* showed instead a significant increase after resveratrol administration.^16^ Similar results were obtained in a study of Hong and co‐workers^17^ in which the polyphenols quercetin aglycone and quercetin monoglycosides, extracted from onion, were used in DSS‐induced colitis female ICR mice. The study demonstrated that DSS treatment reduces *Firmicutes* and increases *Proteobacteria*, but quercetin supplementation can improve these effects, even at low dose (0.21–0.36% of the diet). Chlorogenic acid is the predominant form of the phenolic acids, which is found in coffee; a cup of coffee contains from 27.33 to 121.25 mg of chlorogenic acid.^18^ Evidence from experimental and epidemiological studies indicates that chlorogenic acid can promote a broad range of health benefits^19^ and showed an effective growth inhibitory effect against certain pathogenic bacteria.^20^ Caffeic acid is a hydrolysed metabolite of chlorogenic acid by mucosal and/or microbial esterase in the intestinal tract^21^ and it can reverse the dysbiosis present in colitis. In a study,^22^ colitis was induced in female C57BL/6 mice by administration of 2.5% (w/v) DSS after caffeic acid administration (1 mM). Results showed that caffeic acid exerted anti‐inflammatory effects in DSS colitis mice, the ratio of *F/B* increased in DSS colitis mice, but caffeic acid could inhibit this effect; moreover, caffeic acid increased the relative abundance of *Akkermansia* in DSS.

Dietary polyphenols have been associated with a reduced risk of developing obese‐related chronic diseases, including type 2 diabetes, cardiovascular diseases, nonalcoholic fatty liver disease, and some cancers.^23^ Accumulating evidence indicates that the gut microbiome influences metabolic health; however, limited information is available on how these polyphenols affect the gut microbiota and lipid metabolism. High‐fat diets (HFDs) are considered as perturbing factors of the gut microbiota profile: in general, a higher ratio of *F/B* is observed in obese than lean individuals. The studies analyzed below focused on the modulatory effects of polyphenols on the gut microbiota and on their anti‐obesity mechanisms. Red raspberries, a rich source of anthocyanins, ellagic acid, and epicatechin, have been extensively studied for their ability to prevent or treat diet‐induced obesity. At this purpose, the study of Xian and colleagues^24^ analyzed the effects of polyphenolic extracts isolated from whole fruit, pulp, or seed of red raspberries on the gut microbiome of obese mice. C57BL/6 male mice were fed for 16 weeks with low fat diet (LFD, 10% of fat), HFD (45% of fat), HFD and 0.4% whole fruit polyphenols (HFDwhole), HFD and 0.1% seed polyphenols (HFDseed), and HFD and 0.3% pulp polyphenols (HFDpulp). The polyphenol content of the various extracts was different, the pulp contained predominantly anthocyanins (37% of total polyphenols' content, TPC), seeds contained ellagic acid (40.7% of TPC), while whole fruit contained ellagic acid (23.7% of TPC) and epicatechin (27.0% of TPC). The analyses carried out on the stools showed diet‐associated changes in the gut microbiota, at the genus level compared to the LFD: the HFD significantly decreased the abundance of *Bifidobacterium*, but the HFDseed increased the amount of *Bifidobacterium* to levels higher than those found in LFD. HFD also decreased the levels of *Dehalobacterium*, *Bacteroides*, and unclassified *Lachnospiraceae* compared to LFD. The levels of the *Dehalobacterium* were further decreased in mice fed HFDwhole and HFDpulp compared to HFD. In HFD mice an increase of *Dorea*, *Ruminococcus*, and an unclassified genus from *Mogibacteriaceae* was observed. Conversely, HFDseed decreased the levels of *Ruminococcus* and *Mogibacteriacea* and HFDpulp lowered specifically *Mogibacteriacea*. Finally, pulp and seed polyphenols increased *Roseburia* compared to HFD. The differential effects on the gut microbiota may have been influenced by polyphenolic profiles found in whole fruit, pulp, or seed. Blueberries are also high in phenolic compounds, especially anthocyanins. Blueberry polyphenol extract changed the composition of the gut microbiota in C57BL/6J mice HFD‐fed. The HFD group, at the phylum level, had higher abundance of *Firmicutes*, *Actinobacteria*, and lower abundance of *Bacteroidetes*, *Spirochaetes*, *Cyanobacteria*, and *Tenericutes*. Blueberry extract increased the levels of *Proteobacteria* and *Deferribacteres* and decreased the level of *Actinobacteria*. At genus level, HFD increased *Allobaculum* and decreased *Lactobacillus*, *Ruminococcus*, *Adlercreutzia*, *Prevotella*, *Flexispira*, and *Streptococcus*; while blueberry extract increased *Bifidobacterium*, *Desulfovibrio*, *Adlercreutzia*, *Helicobacter*, and *Flexispira* and decreased *Prevotella*. Blueberry polyphenols can modulate the gut microbiota enhancing the concentration of specific beneficial bacteria.^25^ Similar results with blueberry were obtained using whole polyphenol extracts separated in three fractions: anthocyanins and phenolic acids; oligomeric proanthocyanidins and phenolic acids and flavonols; proanthocyanidins polymers. In this study, the effects on cardio‐metabolic parameters and gut microbiota in C57BL/6J male mice high‐fat high‐sucrose‐diet (HFHSD) were evaluated and the polyphenolic fractions responsible for the observed effects were determined. The results showed an improvement of glucose tolerance after the administration of whole‐extract polyphenols and compared to HFHSD; whole‐extract increased the proportion of *Adlercreutzia equolifaciens*, whereas oligomeric proanthocyanidins and phenolic acids fraction increased the proportion of *Akkermansia muciniphila*.^26^

Peach is another fruit rich in polyphenols, in particular peach peel contains many of them, and a study analyzed the effects of peel peach extracted polyphenols (PPEP) on mice fed HFD.^27^ Female ICR mice have been divided in the following groups: control group, CTR; HFD (60% calories from fat); HFD with 150 mg kg^−1^ BW PPEP; and HFD with 300 mg kg^−1^ BW PPEP. PPEP significantly inhibited the lipid accumulation in HFD‐fed mice, and it regulated the composition of the gut microbiota. Indeed, the abundances of *Lactobacillus*, *Bacteroides*, *Lachnospiraceae*, *Prevotellaceae*, *Alloprevotella*, *Akkermansia*, *Roseburia*, and *Ruminococcus* were increased in HFD‐PPEP mice compared to HFD alone mice. The administration of PPEP exhibited a significant decrease in the ratio of *F*/*B*, while HFD mice showed a significant increase in this ratio. PPEP could regulate the diversity and richness of the intestinal microbial communities. Polyphenols extracted from citrus fruits, as neohesperidin, have beneficial effects on obesity and intestinal microbiota. Neohesperidin administration attenuated the weight gain, the inflammation, and insulin resistance in mouse HFD‐feed, and it reversed HFD‐induced intestinal microbiota dysbiosis. Through 16S rRNA gene sequencing it was demonstrated that, at order level, HFD increased the relative abundance of *Lactobacillales* and reduced the abundance of *Bacteroidales*, whereas neohesperidin reversed these alterations. Compared to the HFD‐fed mice, neohesperidin decreased the proportion of *Faecalibaculum* and increased *Blautia*, *Mucispirillum*, *Lachnospiraceae_UCG‐006*, *Streptococcus*, *Enterorhabdus*, and *Bacteroides*.^28^ Comparable results were obtained using extracts rich in proanthocyanidins. Indeed, the extracts obtained from *Pyracantha fortuneana* fruit (PFE), particularly rich in proanthocyanidins (116.3 ± 13 mg g^−1^ dry weight), were administered (4% of diet) to male Sprague Dawley rats in combination with HFD (12% kcal in fat) for 8 weeks. PFE intervention ameliorated HFD‐induced intestinal barrier dysfunction and improved protein expression levels of tight junction proteins. Several bacterial groups, which were linked with gut barrier integrity, were modulated after PFE administration; at family level, the abundance of *Actinobacteria*, *Bacteroidaceae*, *Corynebacteriaceae*, and *Lactobacillaceae* was increased compared to HFD group, and the HFD‐induced increase in *Ruminococcaceae*, *Helicobacteraceae*, and *Desulfovibrionaceae* were abolished.^29^ Liu and colleagues^30^ analyzed the anti‐inflammatory effects and modulation of the gut microbiota of grape seed proanthocyanidin extract (GSPE) in HFD‐induced obesity. Male C57BL/6 mice were fed a normal diet and a HFD with or without GSPE (300 mg kg^−1^ BW at day) by oral gavage for 7 weeks. GSPE decreased plasma levels of inflammatory cytokines and epididymal fat mass, and improved insulin sensitivity. Compared to HFD group, the abundance of *Firmicutes* was decreased in the HFD + GSPE, while the abundance of *Proteobacteria* was increased. In addition, at the genus level, GSPE supplementation enhanced the abundance of *Clostridium XIVa*, *Roseburia spp*., and *Prevotella*; these bacteria species improved host metabolic perturbations due to HFD feeding. Furthermore, the supplementation of catechins to high‐fat fed mice was found to have a significant impact on the gut microbiota. Male C57BL/6J mice were fed a HFD containing 2% of catechin‐rich green tea extract (GTE, containing 48% of EGCG) for 8 weeks. HFD increased the levels of *Firmicutes*, whereas GTE lowered this; at phylum level, *Actinobacteria* and *Verrucomicrobia* were increased in response to GTE. At the order level, HFD increased the abundance of *Clostridiales* and decreased *Bacteroidales*, *Bifidobacteriales*, *Verrucomicrobiales*, and *Turicibacterales*, while GTE decreased *Clostridiales* and increased *Bacteroidales*, *Bifidobacteriales*, *Verrucomicrobiales*, and *Turicibacterales*. In addition to improving intestinal dysbiosis, GTE decreases the effects of HFD, decreasing the metabolic disorders associated with obesity.^31^ This article confirmed the results obtained by Wang and co‐workers^32^ where the effects of HFD and tea polyphenols on gut microbiota and lipid metabolism had been investigated. Overall, the results showed that HFD significantly impacted gut microbiota, increasing *F*/*B* ratio, but this ratio could be reduced by tea polyphenols administration, together with a reduction in BW gain and levels of serum lipids. Another compound able to reverse HFD‐induced dysbiosis is epigallocatechin 3‐*O*‐(3‐*O*‐methyl) gallate (EGCG3‐Me) presented in oolong tea. The EGCG3‐Me treatment in germ‐free C57BL/6J mice, colonized by human fecal microbiota, significantly increased the relative abundance of *Bacteroidetes* and *Proteobacteria* and decreased the relative abundance of *Firmicutes*.^33^

Quercetin is a polyphenol with a wide range of biological activities, and it was also used for mitigating the onset and development of atherosclerosis; recent studies have highlighted the role of the gut microbiota in atherosclerosis. This study examined the effects of quercetin administration (100 μg per day) in LDLR^−/−^ C57BL/6 mice HFD‐fed (LDL receptor‐deficient mouse LDLR^−/−^ is a hypercholesteraemic mouse model). Quercetin supplementation reduced BW gain, atherosclerotic lesions, inflammatory responses, and oxidative stress. Analysis of the gut microbiota revealed that at phylum level the relative abundances of *Actinobacteria* and *Bacteroidetes* were raised and *Firmicutes* was reduced. At the genus level, *Akkermansia*, *Bacteroides*, *Parabacteroides*, and *Ruminococcus* were increased after quercetin supplementation, while *Lactobacillus* was decreased compared to control group.^34^ Similarly, resveratrol has been used as a remedy for atherosclerosis^35^; indeed, resveratrol attenuated trimethylamine‐N‐oxide (TMAO)‐induced atherosclerosis in ApoE^−^/^−^ mice, decreasing TMAO concentration by modulation of the gut microbiota producing trimethyl‐amine. Analysis of microbiota by qPCR assay showed that resveratrol increased the abundance of *Bacteroidetes* and decreased *Firmicutes* compared to the control group. At genus‐level, resveratrol induced an increase of *Bacteroides*, *Lactobacillus*, *Bifidobacterium*, and *Akkermansia*, instead *Prevotella*, uncultured *Ruminococcaceae*, *Anaerotruncus*, *Alistipes*, *Helicobacter*, and uncultured *Peptococcaceae* were decreased, all bacteria associated with high plasma TMAO concentrations. Rutin (a glycoside combining quercetin and the disaccharide rutinose) is a flavonoid found in many plants, poorly absorbable in the intestine, but an excellent substrate for the gut microbiota. Rutin supplementation reduced the number of *Firmicutes*, especially *Lachnospiraceae* family, and promoted the growth of *Proteobacteria* phylum compared to male C57BL/6J mice HFD‐fed.^36^ Many other phenolic compounds have been used as possible modulators of the gut microbiota, and all of them have restored the dysbiosis induced by HFD. The use, for example, of chlorogenic acid (150 mg kg^−1^ per day) in ICR male mice HFD‐fed reversed the dysbiosis, inhibiting the growth of *Desulfovibrionaceae*, *Ruminococcaceae*, *Lachnospiraceae*, *Erysipelotrichaceae*, and raising the growth of *Bacteroidaceae*, *Lactobacillaceae*, and improving the lipid profile.^37^ *Cyclocarya paliurus* (*Batal*.) *Iljinskaja* is an edible and medicinal plant that contains abundant number of flavonoids, among which the most present is kaempferol. Administration of the extract from *Cyclocarya paliurus* ameliorated the obesity‐induced gut dysbiosis in male HFD‐fed C57BL/6J mice; stimulated the growth of *Prevotellaceae* and *Bacteroidaceae* and it decreased the growth of *Eubacteriaceae*, *Lachnospiraceae*, *Ruminococcaceae*, and *Clostridiaceae*.^38^

The dysbiosis HFD‐induced, in addition to altering lipid metabolism leading to the onset of obesity, and it leads to increased risk factors for metabolic syndrome resulting in type 2 diabetes and hypertension; in addition, gut dysbiosis HFD‐induced can give neuroinflammatory processes.^39^ HFD can increase intestinal permeability, facilitating the passage of lipopolysaccharide (LPS) into the circulation. LPS may activate CD14/TLR4 (Toll like receptor 4) signaling in target tissues, such as brain, and modify the blood–brain barrier inducing neuroinflammation. Anthocyanins found in berries, exhibit anti‐neuroinflammatory properties in the context of obesity.^40^ Marques and colleagues^41^ have shown that the administration of blackberry anthocyanin‐rich extracts (25 mg kg^−1^ BW per day) modulates gut microbiota and alters tryptophan metabolism, increasing the production of the neuroprotective metabolite kynurenic acid. In HFD‐fed Wistar rats, the abundance of *Akkermansia*, *Prevotella*, *Oscillobacter*, *Ruminococcus*, and *Paraeggerthella* was decreased and the genera *Desulfovibrio*, *Rothia*, *Blautia*, *Erysipelatoclostridium*, *Streptococcus*, and *Enterohabdus* were increased. The administration of blackberry anthocyanin‐rich extract increased *Oscillobacter*. Several intervention studies have provided evidence for an improvement in obesity‐associated alterations, including glucose and lipid metabolism, as well as endothelial and cardiac functions due to the beneficial effects of polyphenols, these effects also involve the gut microbiota. Cranberry is an important source of phytochemicals, especially polyphenols, and high polyphenol content is related to ameliorated dyslipidaemia, hyperglycaemia, and oxidative stress in individuals with the metabolic syndrome.^42^ The potential health effects are related to the modulation of the gut microbiota, indeed cranberry extract administration, decreased diet‐induced weight gain, and improved insulin sensitivity in HFHSD‐fed C57BL/6J mice; these effects are related to a shift in the gut microbiota due to increased relative abundance of *Akkermansia*.^43^ Camu Camu (*Myrciaria dubia*) is another fruit rich in flavonoids such as ellagic acid, ellagitannins, and proanthocyanidins. The treatment of male HFHSD‐fed C57Bl/6J mice with 200 mg kg^−1^ of extract of camu camu prevented weight gain and improved glucose homeostasis decreasing the abundance of *Lactobacillus spp*. and promoting the expansion of *Barnesiella spp*., *Turicibacter spp*., and *Akkermansia muciniphila*.^44^ Also, polyphenol extracts from *Smilax china L*. rhizome improved glucose tolerance and reduced body weight, inflammation, and lipid concentrations in serum and liver in HFHSD‐fed C57BL/6J mice. These extracts, rich in neochlorogenic acid and quercetin, decreased *F/B* ratio, relative abundance of *Desulfovibrionaceae*, *Lachnospiraceae*, *Streptococcaceae*, and increased the relative abundance of *Akkermansiaceae* compared to HFHSD group.^45^

Nonalcoholic fatty liver disease (NAFLD) is a condition of chronic liver disorder associated with metabolic syndromes such as obesity, insulin resistance, and diabetes, and several lines of evidence suggests that gut dysbiosis could be related to NAFLD. The spectrum of NAFLD ranges from simple hepatic steatosis, commonly associated with obesity, to nonalcoholic steatohepatitis (NASH), which can progress to fibrosis, cirrhosis, and hepatocellular carcinoma. *Proteobacteria* phylum is involved in hepatic fibrosis and the presence of *Bilophila wadsworthia* can amplify the inflammatory response in the HFD‐induced hepatic pathology.^46^ As already mentioned above, EGCG can modulate the gut microbiota in HFD‐fed mouse^31^; a similar protective effect can be observed in NAFLD where EGCG reduced liver injury, and thus protected against liver steatosis. Indeed, Ning and co‐workers^47^ observed that EGCG improved gut microbiota dysbiosis in male C57BL/6J mice with methionine−choline deficient (MCD) diet‐derived NASH. At the phylum level, the relative abundance of *Bacteroidetes* in the MCD group was decreased, but EGCG supplementation increased *Bacteroidetes* and decreased *Firmicutes*. At genus level, *Alloprevotella*, *Bifidobacteria*, and *Lactobacillus* were increased after EGCG supplementation, *Alistipes*, *Anaerotruncus*, and *Desulfovibrio* were decreased. In male HFD‐fed C57BL/6N mice, the 0.32% daily EGCG administration for 8 weeks, increased the abundance of the phyla *Verrucomicrobia* and *Actinobacteria*, and the genera *Adlercreutzia*, *Akkermansia*, and *Allobaculum*, whereas decreased the phyla *Deferribacteres*, *Proteobacteria*, and *Firmicutes* and the genus *Desulfovibrionaceae* compared to HFD group without EGCG. EGCG inhibited the weight gain, hepatic lesions' size, and triglycerides' content in the liver induced by HFD.^48^ Similar healthy effects against NAFLD were also observed by the polyphenols of *Lonicera caerulea L*. berry (also known as honeyberry). Supplementation with these polyphenol extracts decreased significantly the levels of proinflammatory cytokines and decreased the *F/B* ratio, and the relative abundance of the genera *Staphylococcus*, *Lactobacillus*, *Oscillospira*, and *Ruminococcus*. Furthermore, this supplementation increased the genera *Bacteroides* and *Parabacteroides*.^49^ Resveratrol has been shown to reduce the risk of NAFLD by reducing inflammation and controlling hepatic lipid metabolism.^50^ The administration of resveratrol alleviated NAFLD in HFD‐fed male C57BL/6J mice and modulated the gut bacteria composition; *Desulfovibrio*, *Lachnospiraceae_NK4A316_group* and *Alistipes* were decreased and SCFAs‐producing bacteria *Allobaculum*, *Bacteroides* and *Blautia* were increased.^51^ Like resveratrol, also quercetin supplementation in HFD‐fed mice with NAFLD caused a great impact on gut microbiota composition, reducing *F/B* ratio, and reducing insulin resistance and intrahepatic lipid accumulation. At genus level, quercetin reduced *Desulfovibrio* and increased *Flavobacterium*, *Allobaculum*, and *Sutturella*.^52^ In addition to NAFLD, there are other liver diseases associated with excessive alcohol consumption and HFD exacerbates the liver steatosis caused by this excessive consumption. Park and co‐workers^53^ have shown that polyphenols extracted from mulberry can prevent or delay the effects of ethanol‐induced hepatic steatosis in male HFD‐fed Sprague Dawley rats, modulating gut microbiota. Mulberry extract prevented liver cell destruction by reducing malondialdehyde content in alcohol‐fed rats, decreased plasma triglycerides, and increased glycogen stores. Alcohol intake increased the *Firmicutes* and decreased *Bacteroidetes* population, this ratio was lowered by mulberry administration. In alcohol group, the *Bacteroidales* content was lowered, but mulberry increased the abundance of this order; moreover, *Desulfovibrionales* and *Campylobacterales* were higher in the mulberry group than in the alcohol group. *Lactobacillales* were elevated by mulberry but not by alcohol intake. Similarly, polyphenols extracted by vinegar regulated gut microbiota composition and ameliorated ethanol‐induced liver injury in ICR male mice. Ethanol administration caused an increase in *F/B* ratio, but vinegar polyphenols decreased this ratio. At the genus level, the relative abundances of *Akkermansia*, *Lachnospiraceae_NK4A136_group*, and *Bacteroides* were decreased in the ethanol group, whereas the same were increased by vinegar treatment. These results indicate that vinegar polyphenols reversed ethanol‐induced gut dysbiosis.^54^

#### 2.1.2 Animal studies about pure polyphenols' effects on pre‐established dysbiosis

As previously said, in the studies analyzed above, the administration of polyphenols occurred in conjunction or shortly before the administration of the pathology inducer, as HFD for obesity, or DSS for IBDs. For this reason, instead of analyzing the effects of polyphenols on a pre‐existing dysbiosis, the preventive action of polyphenols on the onset of dysbiosis was evaluated. On the contrary, in the studies analyzed below, the dysbiosis is first induced and then the effects of polyphenols are observed, so analyzing their possible effects on pre‐established dysbiosis. The study by Zheng and colleagues^55^ is one of the few in which the administration of polyphenols occurs after the induction of the disease; indeed, the mice were fed for 8 weeks with HFD and afterward they were fed with the HFD plus procyanidin for an additional 12 weeks. The consumption of procyanidins and similar compounds has improved parameters related to HFD‐induced obesity such as body weight, lipid profile, and the composition of the gut microbiota. The administration of 100 mg kg^−1^ procyanidins for 12 weeks to male C57BL/6J mice HFD‐fed led to an increase in the abundance of *Bacteroidetes* reducing *F/B* ratio. Procyanidins treatment increased the genera *Rikenellaceae* RC9, *Blautia*, *Anaerotruncus colihominis*, *Helicobacter hepaticus*, and decreased the genera *Rikenella*, *Lachnospiraceae*_FCS020_group, *Lachnospiraceae*_UCG‐006, *Desulfovibrio*.^55^ Supplementation with procyanidins modified the gut microbiota composition, reducing genera such as *Lachnospiraceae*, linked to the development of obesity and type 2 diabetes.^56^ Zhao and co‐workers^61^ evaluated if a combination of quercetin and resveratrol could restore the dysbiosis induced by HFD. This study, like that of Zheng,^56^ previously mentioned, analyzed the effects of polyphenols only after the induction of the disease. Wistar rats were treated with a HFD for 2 weeks and subsequently were divided into two groups: HFD and HFD plus a mixture of resveratrol (15 mg kg^−1^ BW per day) and quercetin (30 mg kg^−1^ BW per day) for the following 10 weeks. After 10 weeks, quercetin and resveratrol decreased *Firmicutes* together with the reduction of the body weight, serum lipids, and inflammatory cytokines. At the family level, quercetin and resveratrol decreased the relative abundance of *Lachnospiraceae*, *Desulfovibrionaceae*, *Acidaminococcaceae*, and *Coriobacteriaceae* families, all of which were increased after 12 weeks of HFD; and increased *Ruminococcaceae*, *Bacteroidales*_S24‐7_group, and *Christensenellaceae*. At the genus level, a higher abundance of *Akkermansia* and reduction in *Lachnoclostridium* and *Bilophila* in the quercetin and resveratrol group was observed compared to HFD. These results indicated that administration of quercetin and resveratrol can improve HFD‐induced obesity and reduce gut microbiota dysbiosis, confirming the work conducted by Etxeberria and co‐workers^57^ where treatment with quercetin (30 mg kg^−1^ BW per day) attenuated the increase in *F/B* ratio in rats HFD + sucrose‐feed, decreased the bacterial species associated to diet‐induced obesity (*Erysipelotrichaceae*, *Eubacterium cylindroides*, and *Bilophila wadsworthia*) and increased some that have been inversely related to obesity (*Bacteroides vulgatus*, *Akkermansia muciniphila*). Moreover, seaweed polyphenols extracted from brown macroalga *Lessonia trabeculate* decreased blood glucose levels, serum lipid profiles, and improved insulin resistance in diabetic C57BL/6J mice by the modulation of the gut microbiota. Male C57BL/6J mice HFD‐fed for 4 weeks were injected intraperitoneally with 40 mg kg^−1^ streptozotocin (STZ) to induce diabetes. Subsequently diabetic rats were divided into two groups, one group received daily 200 mg kg^−1^ of polyphenol‐rich extracts by gavage, whereas another group received saline solution for 4 weeks. The 16S rRNA gene high‐throughput sequencing showed that polyphenol extract administration decreased the *F/B* ratio compared to diabetic control mice, at the genus level the relative abundance of *Odoribacter*, *Muribaculum*, *Alistipes*, and *Parabacteroides* were increased.^58^ Zhang and colleagues^62^ analyzed the effects of green tea polyphenols on intestinal microbiota and anti‐obesity mechanisms. 6‐week‐old male C57BL/6J mice were colonized with gut microbiota of healthy volunteers and HFD‐fed for 7 days. For the next 8 weeks mice were divided in groups: HFD group and HFD + GTE (0.1% w/w). Green tea polyphenols decreased at family level the abundance of *Eubacteriaceae*, *Lachnospiraceae*, *Ruminococcaceae* and *Clostridiaceae*; and increased the abundance of *Prevotellaceae* and *Bacteroidaceae*. GTE alleviated HFD‐induced inflammation in liver.

The animal models used in the studies here analyzed help to understand the biological effects that polyphenols could determine on the gut microbiota dysbiosis, but caution is required in the extrapolation of these results for humans, because it has been seen that most of the genera and bacterial species present in mice are not found in humans, although the gut microbiota of mice and humans belong to the same bacterial phyla.^63^ Moreover, the analyzed studies use different experimental models, male vs. female mice, and for this reason the results could be different as the composition of the intestinal microbiota is different between the genders. Finally, the above literature analysis indicates that more studies are needed in animal models about the polyphenols' actions on overt metabolic pathologies to understand their putative healthy action against pre‐established dysbiosis.

### 2.2 Human intervention studies

As discussed above, dietary polyphenols have been associated with a variety of health benefits related to dysbiosis‐associated chronic‐metabolic‐inflammatory diseases in animals, although fewer studies have been carried out in relation to pre‐existing dysbiosis. On the contrary, human clinical trials on the effects of polyphenols on dysbiosis‐associated disease are limited, but considering that patients with overt pathologies were enrolled, all the trials included an analysis of polyphenols actions on pre‐existing dysbiosis. In a randomized, double‐blind, placebo‐controlled study the effects of polyphenol supplementation on the gut microbiota of overweight and obese men and women (BMI = 29) were investigated. Subjects received a combination of EGCG and resveratrol (282 and 80 mg day^−1^, respectively) or placebo for 12 weeks. Analyses of bacteria phyla were conducted with real‐time PCR assays. Abundance of *Bacteroidetes* was higher in men as compared to women, whereas *Gammaproteobacteria*, *Actinobacteria*, and *Firmicutes* were comparable. The administration of EGCG and resveratrol decreased the relative abundance of *Bacteroidetes* in men but not in women. *Firmicutes*, *Actinobacteria*, *Gammaproteobacteria*, *Akkermansia muciniphila*, sulphate‐reducing bacteria, and acetogenic bacteria were not affected by administration neither in men nor in women. A higher abundance of *Bacteroidetes* in men has been related to an unhealthier metabolic profile (i.e., increased fasting glucose and inflammatory markers) as compared with women.^64^ Several studies have demonstrated in humans gender differences in gut microbiota, probably explained by differences in sex hormones.^65^, ^66^ Finally, in this study real‐time PCR was used to analyze the composition of the intestinal microbiota, focusing only on some bacterial strains, and probably sequencing would have led to more specific results.^64^ In another trial, 8 weeks of daily supplementation of 215 mg of anthocyanins and 2.7 g of prebiotic fibers in male and female volunteers with obesity (BMI ≥34) resulted in a decrease of *Firmicutes*, *Actinobacteria*, and *F*/*B* ratio for the increase of *Bacteroidetes*, but nonsignificant decrease in the inflammatory marker fecal calprotectin and BW were observed. In this study, using sequencing, contrary to the previous one, no differences are observed in the composition of the gut microbiota between men and women, but the analysis is done only at the phylum level; furthermore, it is not possible to differentiate if the results obtained are due to anthocyanins, prebiotics, or the combination of both. Finally, the short study duration could be the reason that no significant changes in inflammatory parameters and BW were observed.^67^ Walker and colleagues^68^ studied the effects of resveratrol on insulin resistance, glucose homeostasis, and concomitant effects on fecal microbiota in insulin‐resistant subjects (Caucasian and non‐Caucasian) with the metabolic syndrome. Obese men were randomized to receive 2 g daily of *trans*‐resveratrol or a placebo for 30 days. *Trans*‐resveratrol in Caucasian subjects showed effects on glucose homeostasis, reducing insulin resistance, and declining the 120‐min glucose concentration and the area under the curve in the oral glucose tolerance test; however, these effects were not observed for non‐Caucasian volunteers. Analysis of fecal samples revealed that resveratrol in both racial groups decreased *Rikenellaceae*, *Ruminococcus*, *Oscillospira*, *Clostridium*, *Alistipes*, *Odoribacter*, and *Butyricimonas* and increased *Gammaproteobacteria*, *Gemellaceae*, *Turicibacter*, and *Atopobium*. In particular, in Caucasians subjects, *Alistipes*, *Collinsella*, *Christensenella*, *Holdemania*, and *Turicibacter* decreased after *trans*‐resveratrol treatment and *Bilophila* increased. In non‐Caucasians, *Ruminococcaceae*, *Alphaproteobacteria*, *Christensenella*, *Odoribacter*, and *Clostridium* decreased, instead *Proteobacteria* increased. At baseline, Caucasian subjects had a higher concentration of *Collinsella*, *Clostridiaceae*, and *Ruminococcus* than non‐Caucasian; but *Streptococcus* and *Lactobacillales* were over‐represented in the non‐Caucasians. In post‐treatment, the Caucasian showed higher levels of *Akkermansia muciniphila*, *Fusobacteria*, and *Megamonas*, compared to the non‐Caucasians. The authors concluded that the differences observed between Caucasians and non‐Caucasian did not depend on the different absorption and/or metabolism of *trans*‐resveratrol, since the plasma concentrations of *trans*‐resveratrol and its metabolite, dihydroresveratrol, were comparable in the two groups. Data on the impact of polyphenols on the gut microbiota dysbiosis and their mechanisms of action in humans are scarce, but from these data it can be deduced that polyphenols and their metabolites could modulate the gut microbiota composition through the stimulation of the beneficial bacteria growth and the inhibition of the pathogenic ones. The differences noted between the various studies depend on several factors, primarily the type of the used polyphenols, the bioavailability of polyphenols which varies depending on their chemical structure; the dosage used; the duration of the administration; the number and gender of enrolled volunteers (male or female), and finally the gut microbiota analysis techniques used.

## 3 EFFECTS OF POLYPHENOL‐RICH FOODS ON THE GUT MICROBIOTA DYSBIOSIS

The effects of specific polyphenols have been studied using purified compounds, but the observed effects could vary if, instead of the single compounds, the food matrix was used. Indeed, the interaction with other nutritional components could modify their bioaccessibility (i.e., their release from the food matrix during the gastrointestinal digestion) and so their interaction with the gut microbiota. Some studies have analyzed the effects of foods rich in polyphenols on the gut microbiota of animals or humans affected by diseases.

### 3.1 Animal model studies

#### 3.1.1 Animal studies on the preventive action of polyphenols‐rich foods on the onset of dysbiosis

The link between the gut microbiota and IBDs has been investigated previously, by analyzing the effects of pure polyphenols on animals in which the disease was induced. Because dietary intervention is a safe alternative to drugs in preventing and managing IBDs, several studies have analyzed the effects of foods rich in polyphenols on the symptoms of IBDs and on the gut microbiota.

The effects of the administration of different tomato varieties on the gut microbiota of mice with DSS‐induced IBDs were analyzed. For this purpose, C57BL/6 male mice were fed with different 1% tomato‐supplemented diets for 2 weeks; colitis was induced starting from day 14 and finally mice were sacrificed at day 29. Indigo, ResTom, and Bronze varieties were used in this study; Indigo is rich in anthocyanins and flavonols, ResTom is rich in stilbenoids, whereas Bronze variety is rich of all the polyphenols of the first two varieties. Bronze‐enriched diets reduced the appearance of intestinal damage induced by DSS. The pyrosequencing of 16S rRNA genes showed that Bronze and ResTom increased the phylum *Bacteroidetes* and decreased *Firmicutes*; this effect, as the authors explain, could be due to stilbenoids, presents in both tomato varieties. *Parabacteroides* was increased in the tomato‐supplemented diets compared with standard diets; *Lactobacilli* were increased in Indigo or Bronze tomato‐supplemented diets (rich in flavonols and anthocyanins) while genera *Blautia* and *Oscillospira* were decreased in all groups compared to standard diet.^74^ In piglets treated with DSS (1 g kg^−1^ BW), the 8% grape seed meal inclusion (rich in polyphenols and fiber) in the control diet for 30 days affected the *F*/*B* ratio, stimulating the growth of *Prevotella* and *Megasphaera*, while reducing the relative abundance of *Roseburia*.^75^ Honey is an important food with antioxidant and anti‐inflammatory effects and rich in polyphenols such as caffeic acid, chlorogenic acid, and rutin. Honey might play a role in relieving ulcerative colitis due to its good anti‐inflammatory effect. Sprague Dawley rats received a daily solution of *Prunella vulgaris* honey (50% in water, w/w) by gavage at 5 g kg^−1^ and after 1 week the acute ulcerative colitis was induced via 3% DSS for 1 week. Sequencing of V3–V4 region of the 16S rRNA gene showed that honey administration decreased *Bacteroidetes* and increased *Firmicutes* compared to DSS group without honey. At the genus level, *Lactobacillus spp*. increased after honey, while *Lachnospiraceae NK4A136* decreased. The honey treatment decreased the disease activity index and mitigated colonic histopathological damage in rats.^76^ In another study, the effects of different constituents of honey are investigated on DSS‐induced colitis in rats. Sprague Dawley rats were treated with DSS, sugars, honey, polyphenols, or SASP (sulfasalazine). In the honey group twice daily 25 g kg^−1^ BW honey were intragastrically administered. The doses of the corresponding effective constituents in the sugar, honey, and polyphenols groups were equal. The results showed that treatments with honey improve oxidative and inflammatory markers, reducing inflammatory cytokines such as IL‐6, TNF‐α, and TGF‐β1. Pyrosequencing analysis at the genus level showed that honey can significantly reduce the population of *Bacteroides*, *Corynebacterium*, and *Proteus* compared to DSS group. Analyzing the other groups, it was seen that the effects of honey on the microbiota are due to the polyphenols present and not to the sugars; moreover, honey and SASP showed a similar shift in microbial community structure.^77^ Also in propolis like in honey, numerous phenol compounds are present. Wang and co‐workers^78^ have shown that dietary supplementation with polyphenol‐rich propolis can protect against DSS‐induced colitis. Male Sprague Dawley rats were treated by gavage for 1 week with Chinese propolis or Brazilian propolis (300 mg kg^−1^ BW) starting 1 week prior to DSS treatment. Chinese and Brazilian propolis had dissimilar polyphenol compositions and different quantities. The results showed that both types of propolis prevented the damage DSS‐induced in colonic tissue and decreased inflammatory molecules. Rats fed with different propolis had distinct bacterial communities, due to the dissimilar chemical profiles of propolis. Chinese propolis increased the diversity and richness of gut microbiota, both propolis reduced *Bacteroides spp*.

Dietary intervention is a safe and promising alternative to drugs in preventing and managing IBDs and as seen in the previous paragraphs, grape polyphenols modulated the gut microbiota.^61^ For this purpose, Li and colleagues^79^ investigated the effects of whole muscadine grapes or dealcoholized wine on the gut microbiota in mice with chronic colitis. Female C57BL/6J mice were fed a control diet, and a control diet supplemented with freeze‐dried muscadine grape (7%, w/w) or wine (5.5%, v/w). After 28 days, chronic colitis was induced by 3% DSS administrated for 7 days, then the DSS treatment was suspended, and the colitis progressed into a chronic state. Mice were sacrificed at the 56th day. Administration of grape or wine reduced weight loss and other symptoms of chronic colitis in mice. DSS decreased the relative abundance of *Firmicutes*, but grape and wine increased the abundance of *Bacteroidetes*; wine decreased *Verrucomicrobia* too compared to DSS treatment alone. At the order level, DSS decreased *Clostridiales* and increased *Erysipelotrichales*, but wine or grape did not ameliorate these changes. At the genus level, DSS increased *Clostridium* (associated with diarrhea in IBDs), but grapes and wine reverted this increase. Abundance of *Roseburia*, *Lactobacillus*, and *Anaerotruncus* (i.e., SCFAs producing bacteria) was decreased in DSS, wine but not grape increased *Roseburia*, and *Anaerotruncus*, whereas *Lactobacillus* was not affected by the administration of wine and grapes. Wine seemed more effective in improving dysbiosis, and this is probably attributable to the different content of polyphenols.^79^

Previously we discussed how the polyphenols extracted from berries are able to modulate the inflammatory response, improve the glycaemic response, and modify the dysbiosis induced by a diet rich in fats and sugars, but also the consumption of berries can influence the intestinal microbiota. Blueberry supplementation can alter the gut microbiota, reduce systemic inflammation, and improve insulin resistance in HFD–fed male Wistar rats. 8‐week administration of HFD with 10% dried blueberry powder altered the intestinal microbiota of rats; it improved systemic inflammation and insulin sensitivity and restored ileal villus height. In the gut microbiota at the phylum level, blueberries decreased *Firmicutes* and *Bacteroidetes* abundance and increased *Proteobacteria* and *Fusobacteria* abundance compared with HFD without the supplementation. Despite a decrease in *Firmicutes*, blueberry supplementation led to an increased abundance of *Bacilli* (class), especially *Lactobacillales*.^80^ Contrary to other previously analyzed papers, in this work no increase in *Firmicutes* was observed; this could be due to the similar fiber content in the control diet and HFD. Similarly, the supplementation of lingonberries resulted in improvement of dysbiosis and in reduction of triglycerides and atherosclerosis in male Apoe^−/−^ mice HFD‐fed or HFD with 44% lingonberry for 8 weeks. Lingonberries at genus level increased *Bacteroides*, *Parabacteroides*, and *Clostridium* while decreased *Mucispirillum* and *Oscillospira*. At phylum level, *Bacteroidetes* was increased in concurrent with a decrease of *Firmicutes* compared to HFD without supplementation.^81^

Tea produced from the leaves of *Camellia sinensis* is one of the most widely consumed beverages in the world, and tea consumption has been identified to have healthy effects. For this purpose, Liu and colleagues^82^ evaluated the anti‐obesity and gut microbiota modulatory effects of three types of tea on the gut microbiota of HFD‐induced obese C57BL/6J mice. The mice received food and water or tea infusions ad libitum for 13 weeks. The results showed that the tea infusions suppressed BW gain and the accumulation of adipose tissue in comparison to HFD alone and decreased the serum glucose and lipids to levels comparable to the control group. All tea infusions increased *Alistipes*, *Lachnospiraceae*, *Akkermansia sp*., and *Rikenella microfusus* and decreased *Allobaculum sp*., *Bacteroides acidifaciens*, *Clostridium leptum*, and *Parabacteroides goldsteinii* compared to HFD alone. In addition, tea infusions increased *Blautia sp*., *Helicobacter ganmani*, *Oscillibacter sp*., and *Anaerotruncus sp*. Tea intervention modulates bacteria such as *Alistipes*, *Rikenella*, *Lachnospiraceae*, and *Akkermansia*, which may promote the production of short chain carboxylic acids, improve the gut barrier function. Likewise, the supplementation of purple‐leaf tea (*Camellia sinensis L*.) rich in anthocyanins prevented the effects of obesity and metabolic disorders and prevented microbial dysbiosis in C57BL/6J mice HFD‐fed with purple tea for 10 weeks. Purple tea increased the diversity of the microbiota and attenuated the rise of the *F*/*B* ratio caused by the HFD; moreover, the families *Barnesiellaceae*, *Ruminococcaceae*, and *Lachnospiraceae*, together with the genus *Barnesiella*, increased compared to HFD alone.^83^ The administration of *Angelica keiskei* juice to HFD‐fed C57BL/6 mice prevented weight gain, decreased fat accumulation, blood glucose, serum lipid levels, and normalized gut dysbiosis. *Angelica keiskei* had various flavonoids, among which coumarins, phenolic acids, chalcones, and terpenoids; chalcones (xanthoangelol and 4‐hydroxyderricin) are considered the major components. The supplementation of 12.5 g kg^−1^ per day of fresh *Angelica keiskei* juice by gavage for 10 weeks increased *Bacteroides* and decreased *Mollicutes RF9*, *Ruminococcaceae UCG‐014*, *Faecalibacterium*, and *Lactobacillus* compared to HFD group.^59^

#### 3.1.2 Animal studies about polyphenols‐rich foods on pre‐established dysbiosis

Unfortunately, as already mentioned above, there are few studies in which the effects of the administration of foods rich in polyphenols on a pre‐established dysbiosis have been examined. Liso and co‐workers^60^ analyzed the effects of Bronze tomato, rich in polyphenols, on the gut microbiota of Winnie mice as a model of spontaneous ulcerative colitis. Bronze is a tomato enriched in three distinct classes of polyphenols: flavonols, anthocyanins, and stilbenoids. The authors used the Winnie mouse model to investigate the therapeutic rather than the preventive potential of the Bronze‐enriched diet. Freeze‐dried tomato control and Bronze were supplemented by addition at 1% (w/w) to a standard rodent diet for 2 weeks. 16S metagenomics analysis have shown that Bronze enriched diet perturbed the microbial composition of Winnie mice, increasing the relative abundance of *Odoribacter* and decreasing the relative abundance of *Bacteroides* species (*B. chinchilla*, *B. rodentium*, *B. xylanisolvens*), *Escherichia albertii*, *Parabacteroides distasonis*, *and Ruminococcus gnavus*. The Bronze diet also induced anti‐inflammatory genes and decreased pro‐inflammatory molecules such as IL‐17A and IFN‐γ.

Also, tea is able to reverse the intestinal dysbiosis present in mice in which diabetes has been induced. The diabetes mouse model was established by intravenous injection of alloxan 45 mg kg^−1^ in female Kunming mice, after 1‐week mice were fed with corn‐starch diet and matcha tea 0.1% or green tea for 30 days. Tea and matcha tea supplementation had beneficial effects on the regulation of blood glucose. Sequencing analysis showed that in diabetic mice, *Bacteroidaceae*, *Helicobacteraceae*, *Ruminococcaceae*, *Enterobacteriaceae*, *Rikenellaceae*, and *Saccharibacteria_genera_incertae_sedis* were increased, while *Lactobacillaceae*, *Prevotellaceae*, *Coriobacteriaceae*, *Verrucomicrobiaceae*, and *Bifidobacteriaceae* were decreased. Tea and matcha supplementation decreased *Bacteroidaceae*, *Ruminococcaceae*, *Helicobacteraceae*, and *Enterobacteriaceae* and increased *Coriobacteriaceae*, *Lactobacillaceae*, *Prevotellaceae*, and *Bifidobacteriaceae*. Thus, tea has beneficial effects on glucose and can reverse the changes in the microbiota due to diabetes induction.^84^

The analyzed studies have indicated that, in some cases, supplemented and purified phytochemicals do not provide the same biological activity as whole foods rich in the same compounds. These differences could be due to the different experimental models used, the different administration times, and the different bioaccessibility that the same polyphenols could have if administered as a pure compound or within a food matrix. Table 1 summarizes modulation of pre‐established dysbiosis by polyphenols (Section 2.1.2) and polyphenols rich foods (Section 3.1.2) in animal model studies.

**TABLE 1:** Modulation of pre‐established dysbiosis by polyphenols and polyphenols rich foods in animal model studies

Experimental model	Polyphenols' treatment (PF) as compound/Food	Time course	Analytical methods		Microbiota outcomes		Pathology‐related outcomes	References
HFD‐fed male C57BL/6J mice	100 mg kg^−1^ BW daily procyanidins	8 weeks HFD + 12 weeks PF and HFD	16S rRNA gene HTS	↑	*Rikenellaceae RC9*, *Blautia*, *Anaerotruncus colihominis*, *Helicobacter hepaticus*	↓	Body weight	[55]
				↓	*Rikenella*, *Lachnospiraceae_FCS020_group*, *Lachnospiraceae_UCG‐006*, *Desulfovibrio*			
HFD‐fed male Wistar rats	15 mg kg^−1^ BW resveratrol and 30 mg kg^−1^ quercetin per daily	2 weeks HFD + 10 PF and HFD	16S rRNA gene pyrosequencing	↑	*Bacteroidales_S24‐7 group*, *Christensenellaceae*, *Akkermansia*, *Ruminococcaceae*	↑	HDL, adiponectin	[61]
				↓	*Desulfovibrionaceae*, *Acidaminococcaceae*, *Coriobacteriaceae*, *Bilophila*, *Lachnospiraceae*, *Lachnoclostridium*	↓	Body weight, cholesterol, LDL, triglycerides, IL6, TNF‐α, insulin, leptin	
HFD‐fed male diabetic C57BL/6J mice	200 mg kg^−1^ daily polyphenols from brown macroalga *Lessonia trabeculate*	5 weeks HFD, 1 week post STZ	16S rRNA gene HTS	↑	*Odoribacter*, *Muribaculum*, *Alistipes Parabacteroides*	↓	Glucose, cholesterol, triglycerides, LDL, insulin	[58]
				↓	*Firmicutes/Bacteroidetes*			
HFD‐fed male C57BL/6J mice	0.1% GTE in HFD	1 week HFD + 8 GTE and HFD	16S rRNA gene HTS	↑	*Prevotellaceae* and *Bacteroidaceae*	↓	Liver inflammation	[62]
				↓	*Eubacteriaceae*, *Lachnospiraceae*, *Ruminococcaceae*, *Clostridiaceae*			
Male Winnie mouse, spontaneous ulcerative colitis model	1% tomato bronze in standard diet	2 weeks PF post ulcerative colitis onset	16S rRNA gene HTS	↑	*Odoribacter*, *Bacteroides chinchilla*, *Bacteroides rodentium*, *Bacteroides xylanisolvens*, *Escherichia albertii*, *Parabacteroides distasonis*, *Ruminococcus gnavus*	↓	IL17A IFN‐γ	[60]
Female Kunming mice, diabetes model	0.1% matcha/green tea in corn starch diet	PF 1 week post alloxan‐induce diabetes and for 30 days	16S rRNA gene HTS	↑	*Coriobacteriaceae*, *Lactobacillaceae*, *Prevotellaceae*, *Bifidobacteriaceae*	↓	Blood glucose	[84]
				↓	*Bacteroidaceae*, *Ruminococcaceae*, *Helicobacteraceae*, *Enterobacteriaceae*			

Abbreviations: GTE, green tea extracts; HFD, high‐fat diet; HTS, high throughput sequencing; PF, polyphenols' treatment; STZ, streptozotocin.

### 3.2 Human intervention studies

Only few human clinical studies have examined the mitigating activities of foods rich in polyphenols in IBDs‐associated dysbiosis. In a recent trial, the effects of mango administration in combination with drugs normally used in the treatment of IBDs on the intestinal microbiota and on specific inflammatory markers of the IBDs' disease were analyzed. In this study, participants received a daily dose of 300–400 g of mango pulp for 8 weeks (mango was rich in quercetin and kaempferol glycosides, phenolic acids, predominantly gallic acid). Mango intake decreased the plasma levels of proinflammatory cytokines such as IL‐8, growth‐regulated oncogene (GRO), and granulocyte macrophage colony‐stimulating factor (GMCSF) and altered the gut microbiota composition. qPCR analysis showed that, at the genus level, mango increased the levels of *Lactobacillus* (leading to the formation of SCFAs), at species level increased *Lactobacillus plantarum*, *Lactobacillus lactis* (involved in the metabolism of gallotannins), and *Lactobacillus reuteri*. These lactobacilli have anti‐inflammatory activity in the treatment of colitis by the downregulation of the TNF‐α and cyclooxygenase‐2 secretion and upregulating IL‐10.^69^ The use of mango could be a therapeutic strategy in reducing intestinal inflammation associated with IBDs.^70^ Obesity is the major risk factor for the metabolic diseases, like type 2 diabetes, fatty liver disease, and cardiovascular disease, and on the other hand, there is a relationship between gut dysbiosis and obesity. As already analyzed in the previous paragraphs, polyphenols can control dysbiosis by also improving systemic inflammation, and foods rich in polyphenols can have the same effects too. In a study on obese women, *Schisandra chinensis* fruits were taken twice per day as juice (100 ml each) for a period of 12 weeks. The results showed that *Schisandra chinensis* decreased fat mass, fasting blood glucose, triglycerides, aspartate aminotransferase, and alanine aminotransferase. The denaturing gradient gel electrophoresis analysis (DGGE) of the gut microbiota showed that *Akkermansia*, *Roseburia*, *Bacteroides*, *Prevotella*, and *Bifidobacterium* increase, whereas *Ruminococcus* decreased in the treated group compared to the placebo group.^71^ Likewise, the use of red wine polyphenols can be useful for the prevention of cardiovascular and metabolic alterations associated with obesity. In the study of Moreno‐Indias and colleagues, metabolic syndrome patients (Mets) or healthy subjects consumed red wine and dealcoholized red wine (272 ml day^−1^) for 30 days. After red wine and dealcoholized red wine intake, the metabolic syndrome markers such as glucose, triglycerides, and total cholesterol had improved. Analysis of the fecal microbiota by PCR‐DGGE showed that in Mets subjects *Proteobacteria* and *Firmicutes* are higher compared to healthy subjects, but after administration of wine and dealcoholized wine no differences were observed between the microbiota profiles of the two groups of volunteers. After wine administration in the Mets patients the *Fusobacteria*, *Bacteroidetes*, *Faecalibacterium prausnitzii*, and *Roseburia* (i.e., a butyrate‐producing bacteria), and *Lactobacillus* were increased, *Firmicutes*, *Clostridium*, and the *Clostridium histolyticum* were decreased.^72^ Moreover, also the grape pomace (dietary fiber 68.2%, polyphenols 29.6%), a waste product from wine processing, can improve the intestinal dysbiosis in subjects with metabolic syndrome. Subjects at cardio‐metabolic risk (with at least two metabolic syndrome factors) were supplemented with a daily dose of 8 g of grape pomace for 6 weeks. Grape pomace was found to reduce insulin levels only in the half of the participants (named responders), tended to reduce the order *Lactobacillales* in both responders and nonresponders, but increased *Bacteroides* only in nonresponders.^73^ In this study there is no evaluation of the microbiota at the species level, quantitative real‐time PCR was used instead of next‐generation sequencing techniques; furthermore, the lack of the placebo, it made impossible to establish whether the observed effects depended on the high fiber or the polyphenol content of the grape pomace. The study of Vetrani and colleagues^85^ evaluated the effects of a diet rich in polyphenols or a combination of polyphenols and long‐chain *n* − 3 polyunsaturated fatty acids on the gut microbiota of individuals at high cardio‐metabolic risk. Individuals with high waist circumference and at least one additional factor of the metabolic syndrome were assigned to one of the dietary interventions for 8 weeks: high polyphenols (2903 mg day^−1^) or low polyphenols (365 mg day^−1^). Polyphenols were provided by decaffeinated green tea and coffee, dark chocolate, blueberry jam, extra‐virgin olive oil, and polyphenol‐rich vegetables. DGGE analysis of the predominant bacteria showed that high diet polyphenols increased the *Clostridium leptum* and reduced *Eubacterium rectale* and *Blautia coccoides*. Changes in *Clostridium leptum* correlated with the changes in early insulin secretion after the oral glucose tolerance test. In this study too, only certain bacterial species were analyzed; therefore, because relative abundances were not calculated, it is not possible to verify whether small changes have occurred in not so abundant species. Unfortunately, due to the low number of clinical trials and the great heterogeneity between the study models, the analyzed populations, and the methods of analysis used, it is not possible to draw firm conclusions. Table 2 summarizes modulation of pre‐established dysbiosis by polyphenols (Section 2.2) and poliphenols rich foods (Section 3.2) in human trials.

**TABLE 2:** Modulation of pre‐established dysbiosis by polyphenols and polyphenols rich foods in human trials

Experimental model	Polyphenols' treatment (PF) as compound/food	Time course	Analytical methods		Microbiota outcomes		Pathology‐related outcomes	References
37 overweight/obese men/women BMI = 29	282 mg/day ECGC+ 80 mg/day resveratrol or placebo	12 weeks	qPCR	↓	*Bacteroidetes* in men	↑	Fat oxidation in man	[64]
46 obese male/female BMI ≥34	215 mg anthocyanins and 2.7 g of prebiotic fibers daily	8 weeks	16S rRNA gene HTS	↑	*Bacteroidetes*	↓	HbA1c	[67]
				↓	*Firmicutes*, *Actinobacteria*			
28 Caucasian and non‐Caucasian man with MetS	2 g daily trans resveratrol or placebo	30 days	16S rRNA gene HTS	↑	*Gammaproteobacteria*, *Gemellaceae*, *Turicibacter*,*Atopobium*	↓	Insulin resistance in Caucasian	[68]
				↓	*Rikenellaceae*, *Ruminococcus*, *Oscillospira*, *Clostridium*, *Alistipes*, *Odoribacter*, *Butyricimonas*			
				↑	*In Caucasian*: *Bilophila*, *Fusobacteria*, *Akkermansia muciniphila*, *Megamonas*			
				↓	*Alistipes*, *Collinsella*, *Christensenella*, *Holdemania*, *Turicibacter*			
				↑	*In non‐Caucasian*: *Proteobacteria*			
				↓	*Ruminococcaceae*, *Clostridium*, *Alphaproteobacteria*, *Christensenella*, *Odoribacter*			
10 man/women with moderate IBDs	300–400 g mango pulp daily	8 weeks	qPCR	↑	*Lactobacillus planctarum*, *Lactobacillus lactis*, *Lactobacillus reuteri*	↓	IL8, GRO, GMCSF	[70]
28 Obese women BMI ≥25	200 ml daily *Schisandra chinensis* juice	12 weeks	PCR‐DGGE	↑	*Akkermansia*, *Roseburia*, *Bacteroides*, *Prevotella*, *Bifidobacterium*	↓	Fat mass, triglycerides, glucose, AST, ALT	[71]
				↓	*Ruminococcus*			
20 Caucasian man with MetS	272 ml daily red wine/dealcoholized red wine	30 days	Sanger sequencing	↑	*Fusobacteria*, *Bacteroidetes*, *Faecalibacterium prausnitzii*, *Roseburia*, *Lactobacillus*	↑	HDL	[72]
				↓	*Firmicutes*, *Clostridium*, *Clostridium histolyticum*	↓	Glucose, triglycerides, cholesterol, CRP	
49 subjects at cardiometabolic risk	8 g grape pomace	6 weeks	q PCR	↑	*Bacteroides* in no‐responders	↓	Insulin in responders	[73]
				↓	*Lactobacillales*			
20 man/women with high waist circumference, and one component of MetS	2903 mg daily polyphenols from green tea, coffee, dark chocolate, blueberry jam, polyphenol‐rich vegetables, and so on	8 weeks	PCR‐DGGE	↑	*Clostridium leptum*	↑	Glucose tolerance	[85]
				↓	*Eubacterium rectale*, *Blautia coccoides*			

Abbreviations: ALT alanine aminotransferase; AST, aspartate aminotransferase; CRP, c reactive protein; DGGE, denaturing gradient gel electrophoresis; GMCSF, granulocyte macrophage colony‐stimulating factor; GRO, growth‐regulated oncogene; HbA1c, hemoglobin A1c; HTS, high throughput sequencing; MetS, metabolic syndrome.

## 4 CONCLUSION

**FIGURE 1:**
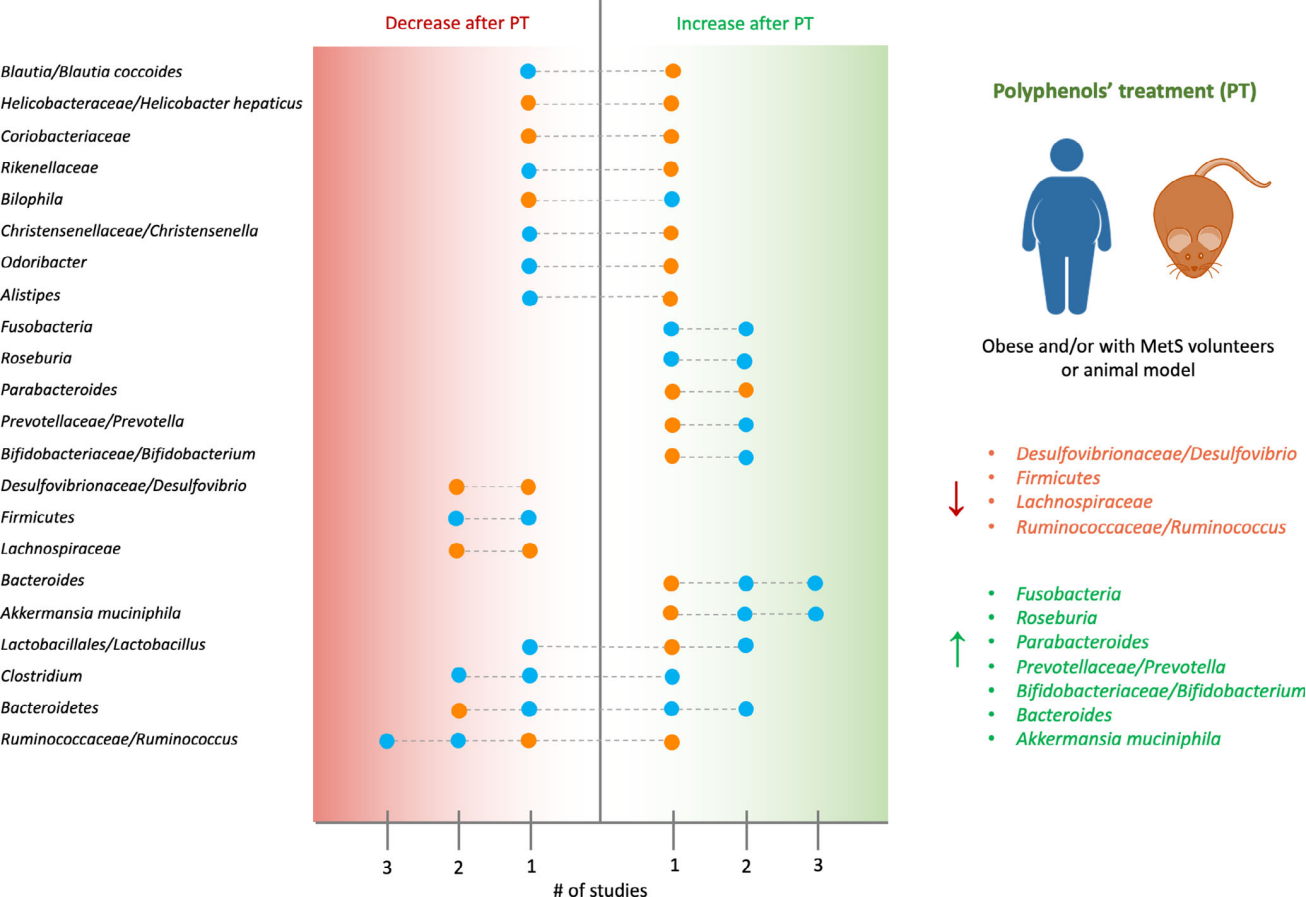
Number of different studies (≥2) that have identified taxa increases or decreases after polyphenols' treatment (PT) in obese and/or with metabolic syndrome (MetS) volunteers or animal models. Orange dot, animal studies; blue dot, human studies. On the right, confirmed changes were summarized

This review has tried to summarize the current knowledge in relation to the modulation of the gut microbiota dysbiosis in several diseases by phenolic compounds and polyphenol‐rich dietary sources. In general, in both animal and humans' studies, polyphenols or polyphenol‐rich foods have shown to influence the disease‐associated dysbiosis, reducing the numbers of potential pathogens/pathobionts, and mainly enhancing the beneficial bacteria, leading to benefits in distinct disorders. Anyway, the main investigated pathological condition in the studies concerning the modulation of pre‐existing dysbiosis by polyphenols was related to cardiometabolic diseases, as obesity and MetS, and the main outcomes are summarized in Figure 1. Although, for many taxa conflicting results were found in relation to the modulatory effect of polyphenols (results that intersect the distribution line of Figure 1), *Desulfovibrionaceae/Desulfovibrio*, *Firmicutes*, *Lachnospiraceae*, and *Ruminococcaceae/Ruminococcus* were found decreased after polyphenols' treatment in both human and animal studies. Similarly, *Fusobacteria*, *Roseburia*, *Parabacteroides*, *Prevotellaceae/Prevotella*, *Bifidobacteriaceae/Bifidobacterium*, *Bacteroides*, and *Akkermansia muciniphila* were found increased after polyphenols' treatment in both human and animal studies. However, the analyzed studies had some limitations: only few studies have examined the impact of the dietary polyphenols on disease‐associated human gut microbiota dysbiosis, and most of them were focused on selected bacterial populations. Furthermore, in animal studies, the administered concentrations of polyphenols (especially for foods rich in polyphenols), were so higher that would be difficult to apply them in humans. In most animal studies, only males were used due to the known resistance of female mice to diet‐induced obesity and insulin resistance; but this limitation precludes the possibility of verifying whether gender differences exist for the benefits of polyphenols against dysbiosis and their potential application to women. Moreover, caution is required in extrapolating the results from animal models to humans because it has been seen that most of the genera and bacterial species present in the animal models are not found in humans, although the gut microbiota of mice and humans belong to the same bacterial phyla. Finally, in most of the studies conducted on animals instead of analyzing the effects of polyphenols on a pre‐existing dysbiosis, the preventive action of polyphenols on the onset of dysbiosis was evaluated. In conclusion, more studies in animal models are needed to understand the putative healthy action of polyphenols against pre‐established dysbiosis; and further well‐structured human trials with larger sample size, different pathologies, longer duration, and high‐throughput molecular techniques, are necessary to provide more conclusive results.

## CONFLICT OF INTEREST

The authors declare no conflict of interests.

## Data Availability

Data sharing not applicable ‐ no new data generated.
